# Association Between Ethylene Oxide Exposure and Complete Edentulism in United States Adults

**DOI:** 10.3390/life15050740

**Published:** 2025-05-03

**Authors:** Yash Brahmbhatt, Michelle Zak, Razan Alhajri, Noura Almulla, Sakeenah Alqallaf, Abdullah Alkandari, Shahad Alsaleh, Hend Alqaderi

**Affiliations:** 1 Tufts University School of Dental Medicine, Boston, MA 02111, USA; 2 Kuwait Ministry of Health, Kuwait City 15462, Kuwait; 3 Department of Public Health, Tufts University School of Dental Medicine, Boston, MA 02111, USA; hend.alqaderi@tufts.edu; 4 Dasman Diabetes Institute, Kuwait City 15462, Kuwait

**Keywords:** ethylene oxide, exposure, complete edentulism, oral health, toxicity, risk factors, environmental toxins

## Abstract

(1) Background: Ethylene oxide (EtO), an environmental pollutant, has been linked to adverse health outcomes through its genotoxic, oxidative stress inducing, and alkylating properties, including potential impacts on oral health. This study explores the association between EtO serum levels and complete edentulism. (2) Methods: Data were analyzed from the 2017–2018 National Health and Nutrition Examination Survey (NHANES) dataset using logistic regression analysis to examine the relationship between EtO serum levels and complete edentulism, adjusting for age, sex, education, race, and diabetes status, and periodontal disease, including a total of 19,225 participants. Of the 19,225 participants, 4933 individuals (25.66%) were completely edentulous, and 14,292 (74.34%) were not. (3) Results: Higher EtO serum levels were associated with increased odds of complete edentulism (OR = 1.61; 95% CI = 1.35–1.93; *p* ≤ 0.001), adjusting for the confounders mentioned above. (4) Conclusions: This analysis of a representative sample of the U.S. adult population showed that individuals exposed to higher levels of EtO had total tooth loss, underscoring the importance of addressing environmental factors in oral health. Further research is needed to understand the mechanism of EtO exposure on oral health.

## 1. Introduction

Missing teeth are not just about a missing smile; they are a potential marker of systemic health issues. Missing teeth have the potential to impact more than just the occlusion, skeletal structure, periodontium structure, and periodontal status [1,2,3,4,5] which can lead to periodontal disease, and environmental factors like ethylene oxide (EtO) may exacerbate these processes, contributing to edentulism. Missing teeth can affect an individual’s nutrition, psychological health, and potentially increase their risk for other systemic diseases [1,2,3,4,5,6,7,8,9]. According to the American College of Prosthodontics (ACP), an estimated 178 million Americans are missing at least one tooth, and around 40 million are completely edentulous [10]. Recent research highlights that missing teeth can be associated with factors for inflammatory conditions such as cardiometabolic diseases, cancers, peripheral neuropathy, and cognitive impairment [11,12,13,14]. Dental caries and periodontal diseases are also common causes of tooth loss, with these conditions frequently acting as precursors to further systemic health issues [15].

Environmental toxins have emerged as another potential contributor to systemic and oral health [16]. Exposure to various environmental pollutants, chemicals, pesticides, and heavy metals in the environment has been shown to potentially exacerbate conditions like periodontal disease and tooth decay [16,17,18,19]. Among various environmental pollutants, EtO has gained particular attention for its health effects, including periodontal disease [20,21]. EtO is a chemical used in sterilization processes and various industrial applications. According to the United States Environmental Protection Agency, products which contain EtO are considered pesticides due to properties which can kill viruses and bacteria [22,23,24,25]. Exposure to high levels of EtO has been associated with various effects like acute toxicity, cancer, neurotoxicity, and respiratory conditions [26]. As a result, the United States Environmental Protection Agency (EPA) has implemented a phased reduction in the OSHA permissible exposure limit for EtO, lowering it from 1 ppm to 0.5 ppm within 3 years, 0.25 ppm within 5 years, and 0.1 ppm within 10 years of the rule’s implementation [22,26].

Given the emerging evidence linking EtO exposure to systemic conditions, we hypothesize a positive association between EtO exposure and complete edentulism, potentially driven by an inflammatory environment. The aim of this study is to examine the relationship between EtO exposure and complete edentulism, using data from the National Health and Nutrition Examination Survey (NHANES) for the years 2017 and 2018.

## 2. Materials and Methods

### 2.1. Study Population

Cross-sectional data from the 2017–2018 cycle of the publicly accessible National Health and Nutrition Examination Survey (NHANES) was used in this study, adhering to the Data Use Guidelines set forth by the National Center for Health Statistics (NCHS) at the Centers for Disease Control and Prevention [27]. Inclusion criteria for this study consisted of individuals who participated in the NHANES 2017–2018 dataset, with complete dental records (specifically data on the presence or absence of complete edentulism), and available EtO exposure data (which were measured through blood tests). Individuals who did not participate in the NHANES 2017–2018 dataset and those who participated in the NHANES 2017–2018 dataset but had incomplete or missing dental records were excluded. This targeted approach is designed to identify potential correlations that could highlight EtO’s impact on oral health and guide preventive strategies.

Since 1999, the NHANES has provided nationally representative data on health conditions, diseases, and associated risk factors in the U.S. This study employs a rigorous methodology, incorporating surveys, laboratory analyses, and clinical assessments to generate comprehensive public health insights [27]. A multistage, stratified, and clustered probability sampling strategy is used by NHANES, ensuring that the collected data are representative of the civilian, non-institutionalized U.S. population.

This study analyzed publicly available NHANES data from 2017 to 2018 and was classified as non-human research, making it exempt from an International Review Board (IRB) review. Nationally representative estimates reflecting the broader U.S. population were obtained by applying survey weights and accounting for NHANES’ complex sampling design. 

### 2.2. Definition of the Dependent Variable: Complete Edentulism

The outcome variable of interest was characterized as individuals with the presence of all missing teeth (complete edentulism/completely edentulous). This variable was identified and coded as a binary where “0” = “not missing all teeth” and “1” = “missing all teeth”, also interpreted as “Having all natural permanent teeth missing, including third molars”. According to the American Association of Oral and Maxillofacial Surgeons (AAOMS), edentulism is defined as the loss of one or more functional teeth, which can be classified as partial edentulism (loss of some teeth) or in this case can be complete edentulism (loss of all teeth); it can be due to factors such as decay, periodontal disease, or trauma [28].

This study focused on individuals with complete edentulism and compared them to those without complete edentulism. This binary classification was used to investigate the association between **complete edentulism** and other variables of interest within a cross-sectional study design.

### 2.3. Description of Independent Variable: Ethylene Oxide

EtO was the primary exposure variable, and it was evaluated by measurements of the chemical compound in participants’ blood serum. This continuous variable was named “LBXEOA” in the NHANES 2017–2018 cohort, and blood samples were collected in vacutainer tubes and processed using standardized laboratory protocols to ensure accurate quantification of EtO exposure [29]. Immediate processing was performed, including centrifugation to separate serum from cellular components and prevent clotting or hemolysis. EtO concentrations were measured in picomoles per gram of hemoglobin (pmol/g Hb)**,** providing a continuous variable for assessing systemic EtO levels in the study population.

### 2.4. Confounding Variables

Age: This variable was defined as the participant’s age at the time of screening and was analyzed as a continuous variable.

Sex: This variable was binary, categorizing individuals as either male or female.

Education: This variable was an ordinal categorical variable representing the level of education completed by participants and is classified into three categories: 0–11 grade, HS/GED, and >HS.

Race: This variable was categorical and classified into the following groups: Mexican American, White, Black, Asian, and Other (including individuals of mixed or other racial/ethnic backgrounds).

Diabetes status: Diabetes status was determined based on participants’ self-reported responses during the NHANES. The survey determined whether a health professional had ever diagnosed them with diabetes or pre-diabetes, with the response categorized as “no”, “yes”, or “borderline” (with “borderline” indicating pre-diabetic), making it a categorical variable.

Gum diseases: This variable was derived from participants’ self-reported responses in the NHANES questionnaire. This variable was assessed based on participants’ responses to the question “Have you ever had treatment for gum disease, such as scaling and root planing, sometimes called ‘deep cleaning’?” The response was recorded as “Yes” or “No”, making it a binary variable.

### 2.5. Statistical Method

A weighted analysis was conducted to analyze the distribution of the categorical variables within the study and t-tests were conducted to analyze the continuous variables. To assess the relationship between complete edentulism and serum levels of EtO (pmol/g Hb), a weighted multivariate binary logistic regression model was utilized, adjusting for potential confounding factors such as age, sex, education, race, diabetes status, and gum disease. The STATA 17 software was utilized in conducting all statistical analyses with a significance threshold of 0.05.

## 3. Results

Table 1 displays a descriptive summary of the categorical variables within the population characteristics, specifically comparing individuals with complete edentulism and those without complete edentulism. Weighted analysis was conducted to calculate the *p*-values. Among the 19,225 individuals in the sample, 4933 (25.66%) were completely edentulous; among these, 85.14% had previous treatment for gum disease (periodontal disease).

Table 2 displays a descriptive summary of the continuous variables in comparison with and without complete edentulism. Individuals with complete edentulism exhibited higher serum EtO levels (95.49 pmol/g Hb) when compared to individuals without complete edentulism (50.88 pmol/g Hb), with a significant *p*-value of <0.001.

Table 3 displays the results from the weighted multiple logistic regression model showing the association between EtO levels and complete edentulism in the United States, 2017–2018. For each one unit (pmol/g Hb) increase in the serum concentration of EtO, the odds of having complete edentulism increased by 61% (OR = 1.61, 95% CI: 1.338–1.925, *p* ≤ 0.001). It was seen that age and education levels were statistically significant covariates in the model, with individuals who were 45–59 and individuals who were above 60 having higher odds of being completely edentulous (OR = 2.92, 95% CI: 1.095–7.779, *p =* 0.034 and OR = 6.65, 95% CI: 2.755–16.04, *p* ≤ 0.001, respectively). Individuals with education greater than high school presented significantly as well (OR = 0.24, 95% CI: 0.151–0.378, *p* ≤ 0.001). Race and diabetes status did not exhibit a significant association within this model (race exhibiting *p*-values of 0.126 for White, 0.265 for Black, 0.045 for Asian, and 0.410 for other, while diabetes exhibited *p*-values of 0.974 for “yes” and 0.637 for “borderline”).

## 4. Discussion

This analysis of a representative sample of the U.S. population demonstrated a positive association between elevated EtO serum levels and complete edentulism. Individuals with higher exposure to EtO exhibited higher rates of total tooth loss. EtO, a flammable and colorless gas commonly used in the manufacturing of various chemicals and in the sterilization of medical devices and food products [30], may therefore represent an environmental factor contributing to oral health outcomes.

EtO can enter the body through inhalation into the lungs, followed by absorption into the bloodstream, or through ingestion via the gastrointestinal (GI) tract [31]. Due to its small molecular size, EtO can bypass respiratory defenses such as nasal hair and macrophages [32]. Tooth loss is most commonly caused by periodontal disease and dental caries, though other factors, such as trauma or lack of access to dental care, also play a role. The link between EtO exposure and complete edentulism observed in this study may be explained by several underlying biological mechanisms. Elevated serum levels of EtO are known to contribute to systemic inflammation [33,34], which can affect multiple organs, including the oral cavity.

Periodontal disease, a major oral inflammatory condition influenced by systemic inflammation, is widely recognized as a leading cause of tooth loss [35,36]. The inflammation and tissue destruction associated with periodontal disease can contribute to the destruction of tooth-supporting structures [37]. Individuals with periodontitis exhibit increased serum levels of inflammatory markers, such as C-reactive protein (CRP) and interleukin-6 (IL-6), suggesting that EtO may exacerbate inflammation. Recent findings indicate that osteoclastic bone damage in periodontitis is driven by the receptor activator of NF-kB ligand (RANKL), which is produced by osteoblastic cells and periodontal ligament cells [37,38,39]. Increased inflammation could elevate RANKL production, promoting osteoclastic bone resorption and potentially worsening periodontal damage.

Dental caries can also lead to tooth loss through bacterial acid production, biofilm formation, and inflammation of the pulp [40]. Dysbiosis of the oral microbiome, particularly species like *Mutans* and *Lactobacillus*, which metabolize fermentable carbohydrates and produce organic acids [40,41,42], can lead to caries. Oral inflammation, driven by systemic inflammation, can potentially alter the oral microbiome and increase the risk of dental caries, another key contributor to tooth loss. EtO exposure may exacerbate both systemic and local inflammation through its systemic effects on immune function and inflammation [41,43]. By altering the host’s immune defense and promoting dysbiosis within the oral microbiome, EtO exposure may promote conditions favorable to caries development [41,44]. 

**Figure 1:**
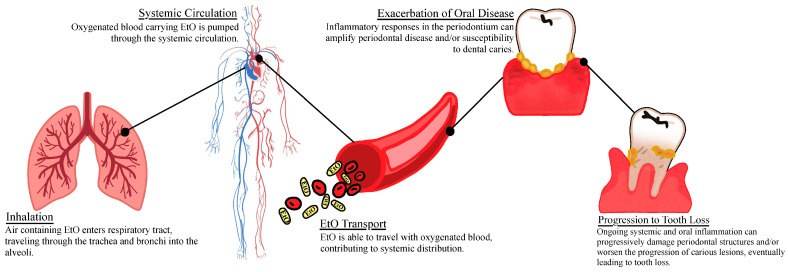
Systemic absorption and oral health impact of ethylene oxide exposure.

Both dental caries and periodontal disease are driven by microbial dysbiosis and inflammatory responses [41,43,44]. While they have an effect on different parts of the tooth and surrounding structure, they both can ultimately cause tooth loss [40,41,42,43,44]. While distinct in their pathology both dental caries and periodontal disease are interrelated. The presence of periodontal disease created an environment of chronic inflammation and deepened periodontal pockets [45]. These deepened periodontal pockets create an environment for acid-producing bacteria which are associated with caries [46,47,48,49]. Similarly, the inflammatory response triggered by dental caries can encourage a cycle of microbial dysbiosis and accelerate both periodontal disease and worsen the progression of the carious lesion (Figure 1).

One other possible underlying biological mechanism linking EtO exposure to complete edentulism is the oxidative damage caused by free radicals [50]. Oxidative stress occurs when an imbalance arises between free radicals and the body’s ability to neutralize them with antioxidants [51]. Once inside, EtO undergoes nucleophilic substitution reactions, producing reactive derivatives that increase oxidative stress, DNA damage, and carcinogenic effects, thus impacting overall health [51,52,53,54,55,56]. Increased serum levels of EtO can create reactive oxygen species (ROS), resulting in cellular damage that can harm gums, soft tissue, and even tooth structure [50,57,58]. This damage may weaken the tooth-supporting bone, cause connective tissue breakdown [50,57], and even lead to enamel degradation [58,59].

### Limitation of the Research

Despite providing critical insights, this study did not specifically exclude individuals based on their health conditions or genetic factors, which could influence EtO metabolism, elimination, susceptibility, or overall vulnerability. Health conditions such as respiratory, metabolic, or hematological disorders may increase susceptibility to EtO exposure [34,54,60]. Genetics also play a significant role in the rate of metabolism and elimination; enzymes such as glutathione-S-transferases (GST) and epoxide hydrolases (EH) help metabolize EtO into less reactive forms [61]. Genetic polymorphisms that reduce GST and/or EH activity may impair detoxification pathways, leading to increased susceptibility to toxic EtO exposure [60,61,62,63,64]. Overall, variations in genetic makeup and underlying health conditions affecting metabolic and repair processes can influence an individual’s ability to manage EtO exposure effectively.

A key limitation of this study was the absence of periodontal examination data and biomarkers indicating oral inflammation. Potential residual confounding factors, such as specific biomarkers, may impact the association between EtO serum levels and complete edentulism. Additionally, while the NHANES provides comprehensive oral health data, it does not capture the duration of edentulism due to its design. Furthermore, the cross-sectional study design limits causal interpretation of the association between EtO levels and complete edentulism. There is a lack on direct studies on EtO and complete edentulism. Prior research has established strong associations between systemic inflammatory markers and periodontitis [35,36,37,38,39], and this study aligns with this body of evidence and extends it by highlighting a potential environmental contributor to these inflammatory processes, connecting it with complete edentulism.

Future studies should aim to understand the mechanistic pathways through which EtO affects oral health, identify and survey populations with increased occupational exposure risk through more detailed variables, and incorporate measures such as the number of years experiencing complete edentulism. These findings suggest that future research should move beyond traditional risk factors for oral health and tooth loss, incorporating a broader perspective that integrates oral and systemic health. Environmental factors, which are often overlooked in oral health research, warrant further investigation to better understand their potential impact on both general and oral health outcomes.

## 5. Conclusions

This study, using a representative sample of the U.S. adult population, identifying a positive association between increased serum EtO levels and complete edentulism. The findings suggest that individuals with higher EtO exposure are more likely to experience complete edentulism. The findings highlight the potential role of environmental factors, such as EtO, in oral health outcomes. The growing recognition of the connection between systemic and oral health suggests that future research should further investigate how environmental exposures like EtO affect both general and oral health, particularly in relation to conditions like complete edentulism.

## Figures and Tables

**Table 1:** Descriptive summary of population characteristics: comparison with and without complete edentulism.

Covariate		Not Missing All Teeth N = 14,292 (74.34%)	Missing All Teeth N = 4933 (25.66%)	Total N = 19,225	*p*-Value
Sex	Male	6963 (48.76%)	2486 (49.52%)	9449 (48.86%)	0.5345
	Female	7329 (51.24%)	2447 (50.48%)	9776 (51.14%)	
Age (in years)	Less than 6	165 (0.58%)	2815 (51.51%)	2980 (7.41%)	<0.001
	6–11	2234 (8.68%)	213 (2.1%)	2447 (7.8%)	
	12–18	2111 (10.73%)	121 (1.09%)	2232 (9.44%)	
	19–44	4345 (37.59%)	455 (10.27%)	4800 (33.92%)	
	45–59	2376 (21.56%)	339 (11.65%)	2715 (20.23%)	
	Above 60	3061 (20.87%)	990 (23.38%)	4051 (21.20%)	
Education	Grade 0–11	1931 (11.76%)	550 (26.13%)	2481 (12.93%)	<0.001
	High School/General Educational Development (GED)	2118 (23.36%)	443 (30.38%)	2561 (23.94%)	
	>High School	5466 (64.88%)	762 (43.49%)	6228 (63.13%)	
Race	Mexican American	4065 (17.49%)	1351 (20.28%)	5416 (17.87%)	0.0017
	White	4474 (60.59%)	1742 (55.22%)	6216 (59.87%)	
	Black	3221 (11.80%)	1023 (12.45%)	4244 (11.89%)	
	Asian	1747 (5.58%)	463 (5.59%)	2210 (5.58%)	
	Other	785 (4.53%)	354 (6.47%)	1139 (4.79%)	
Diabetes	No	12,647 (89.67%)	3737 (88.59%)	16,384 (89.54%)	0.0976
	Yes	1365 (8.43%)	384 (10.01%)	1749 (8.62%)	
	Borderline	273 (1.90%)	58 (1.39%)	331 (1.84%)	
Ever had treatment for gum disease?	No	1037 (23.52%)	137 (14.86%)	1174 (22.74%)	0.0016
	Yes	2858 (76.48%)	689 (85.14%)	3547 (77.26%)	

**Table 2:** Descriptive summary of continuous variables: comparison with and without complete edentulism.

Continuous Variable	Not Missing All Teeth Mean (SD)	Missing All Teeth Mean (SD)	*p*-Value
Concentration of Ethylene Oxide (pmol/g Hb)	50.88 (88.42)	95.49 (129.9)	<0.001

**Table 3:** Multiple logistic regression model: association between ethylene oxide levels and complete edentulism in the United States, 2017–2018.

Covariate		Composite			
		Odds Ratio	Confidence Interval		*p* Value
			Lower	Upper	
Log Ethylene Oxide		1.61	1.338	1.925	<0.001
Age (in years)	45–59	2.92	1.095	7.779	0.034
	Above 60	6.65	2.755	16.04	<0.001
Sex		1.28	0.923	1.788	0.127
Education	High School/General Educational Development (GED)	0.37	0.220	0.614	0.001
	>High School	0.24	0.151	0.378	<0.001
Race	White	1.67	0.850	3.283	0.126
	Black	1.43	0.740	2.773	0.265
	Asian	1.77	1.015	3.075	0.045
	Other	1.42	0.600	3.418	0.410
Diabetes	Yes	0.99	0.617	1.597	0.974
	Borderline	0.79	0.275	2.259	0.637
Ever had treatment for gum disease?		2.07	0.972	4.404	0.058

## Data Availability

The data used in this study are publicly available through the CDC. Additional data supporting the findings can be accessed upon reasonable request by contacting the corresponding author. Any data sharing will comply with privacy regulations and necessary safeguards. For further details, inquiries can be directed to the corresponding author.
